# Surveillance and Correlation of Antibiotic Consumption and Resistance of *Acinetobacter baumannii* complex in a Tertiary Care Hospital in Northeast China, 2003–2011

**DOI:** 10.3390/ijerph10041462

**Published:** 2013-04-09

**Authors:** Jiancheng Xu, Zhihui Sun, Yanyan Li, Qi Zhou

**Affiliations:** 1 Department of Laboratory Medicine, First Hospital of Jilin University, Changchun 130021, China; E-Mail: jianchengxu@yeah.net; 2 Department of Pharmacy, First Hospital of Jilin University, Changchun 130021, China; E-Mails: sunzhihui7777@yahoo.cn (Z.S.); liyanyan0622@yahoo.com.cn (Y.L.); 3 Department of Pediatrics, First Hospital of Jilin University, Changchun 130021, China

**Keywords:** *Acinetobacter baumannii* complex, drug resistance, microbial, antimicrobials susceptibility test, drug monitoring, antibiotic consumption

## Abstract

This study investigated the changes in resistance of *Acinetobacter baumannii* complex and the association of carbapenem-resistant *A. baumannii* complex (CRAB) infection and hospital antimicrobial usage from 2003 to 2011 in a tertiary care hospital in northeast China. *In vitro* susceptibilities were determined by disk diffusion test and susceptibility profiles were determined using zone diameter interpretive criteria, as recommended by the Clinical and Laboratory Standards Institute (CLSI). Data on consumption of various antimicrobial agents, expressed as defined daily dose/1,000 patients/day, were collected retrospectively from hospital pharmacy computer database. Most of 2,485 strains of *A. baumannii* complex were collected from respiratory samples (1,618 isolates, 65.1%), secretions and pus (465, 18.7%) over the years. The rates of antimicrobial resistance in *A. baumannii* complex increased significantly over the years. The rates of CRAB were between 11.3% and 59.1% over the years. The quarterly use of anti-pseudomonal carbapenems, but not other classes of antibiotics, was strongly correlated with the increase of quarterly CRAB (β = 1.661; p < 0.001). Dedicated use of anti-pseudomonal carbapenems would be an important intervention to control the increase of CRAB.

## 1. Introduction

Acinetobacter baumannii complex has emerged as an important pathogen causing a variety of infections, including urinary tract infections, skin and soft tissue infections, and pneumonia and bloodstream infections [1]. *A. baumannii* complex used to be an easy-to-treat pathogen because it was susceptible to a wide range of antibiotic agents [2], however, over the last decade, *A. baumannii* complex has developed various resistance mechanisms to antibiotics [3,4]. The ability to chronically colonize patients and cause outbreaks which are usually hard to eradicate poses significant challenges to infection control and increases healthcare expenditure [5]. The threat of antimicrobial resistance has extended from the hospital setting to the community setting. The trend in increased antimicrobial resistance among bacterial pathogens severely limits the choice of effective antimicrobial agents in both settings. Imipenem and meropenem were traditionally the most effective antimicrobials against *A. baumannii* complex [6], but carbapenem-resistant *A. baumannii* complex (CRAB) has become common worldwide [7]. Due to treatment failure, drug-resistant strains have been associated with higher mortality and prolonged hospital stay compared with susceptible ones. The major risk factors for spread of multidrug-resistant organisms, including CRAB, are poor adherence to infection control measures and overuse of certain antimicrobials [8].

The misuse and overuse of antibiotics is widespread, not only in developing countries, but also in the developed world, and this inappropriate use of antibiotics has led to the rise in antimicrobial resistance. The emergence and spread of antimicrobial resistance is a complex problem that is driven by numerous interconnected factors such as under- or overuse of antimicrobials [9]. However, it is also known that the genetic mechanism used by bacteria to acquire resistance to antibiotics not only promotes their spread within hospital environments, but also confers stability on the resistance genes, even subsequently, in situations of absence of exposure to antimicrobials [10]. In recent years, an increased effort has been directed towards controlling antibiotic use and raising public awareness of the need for prudent use of antibiotics. Over the past decade, many surveillance efforts have drawn attention to this phenomenon [11,12].

This interaction between antibiotic consumption and the development of bacterial resistance to them is of particular interest with regard to *A. baumannii* complex. Given that new therapeutic options for treating infections caused by *A. baumannii* complex with high levels of resistance are not expected to become available in the near future, it becomes imperative to study measures that might be capable of improving the antimicrobial susceptibility of these bacterial agents, thereby making it possible to use the drugs that are available. Studies showing the effects of modifications to antibiotic prescription patterns are especially of interest in relation to combating the emergence of resistance in *A. baumannii* complex [9]. The objectives of this study were to give an overview of changes in antibiotic consumption and resistance of *A. baumannii* complex isolated from a tertiary care hospital in northeast China in nine consecutive years (2003 through 2011).

## 2. Experimental Section

### 2.1. Hospital Setting and Definitions

First Hospital is a teaching hospital affiliated with Jilin University in northeast China. It offers both primary and tertiary referral care. The bed capacities of hospital were 921, 1,084, 1,084, 1,240, 1,731, 1,960, 2,373, 2,572, and 2,898 from 2003 to 2011, respectively. The total numbers of inpatients were 29,323, 36,222, 41,922, 51,185, 59,907, 65,471, 83,172, 94,768, and 110,107 from 2003 to 2011, respectively. The median duration of hospital stays was 12.0, 11.9, 11.5, 10.9, 10.6, 10.1, 10.0, 9.7, and 9.2 days from 2003 to 2011, respectively. Centers for Diseases Control (CDC) criteria were used for the diagnosis of nosocomial infections. Hospital-acquired infection (HAI) was defined as occurrence of infection after hospital admission, without evidence that the infection was present or incubating (≤48 h) on admission.

### 2.2. Bacterial Isolates

For *Acinetobacter* spp., isolates were subcultured to blood agar and McConkey agar plates at this laboratory for purity check and to confirm species identification. Identification was performed using the VITEK 2 system (bioMérieux, Marcy l’Etoile, France) in the microbiological laboratory of the hospital. In addition, conventional biochemical tests including oxidase, Triple Sugar Iron, 42 °C, malonate, and hemolysis on sheep blood agar were used to aid in confirmation of the *A. baumannii* complex. Two thousand four hundred and eighty-five consecutive nonduplicate nosocomial isolates of *A. baumannii* complex were collected during the period from 2003 to 2011 in the hospital. Isolates of the same species from the same patient collected during the same in-patient stay were considered duplicate isolates, and only the first isolate was included from the analysis.

### 2.3. Antimicrobial Susceptibility Testing

*In vitro* susceptibilities of *A. baumannii* complex to 17 antimicrobial agents were determined by the disk diffusion method and susceptibility profiles were determined using zone diameter interpretive criteria, as recommended by the Clinical and Laboratory Standards Institute (CLSI) in 2011 (M100-S21). Breakpoints of cefoperazone/sulbactam were interpreted according to the supplier’s recommendations. Mueller-Hinton agar (Oxoid) was used for all susceptibility tests. The proportion of resistant isolates was calculated by dividing the number of resistant isolates of *A. baumannii* complex by the total number of the isolates tested against the corresponding antibiotic multiplied by 100. *Escherichia coli* ATCC 25922, *Escherichia coli* ATCC 35218, *Klebsiella pneumoniae* ATCC 700603, and *Pseudomonas aeruginosa* ATCC 27853 were used as quality control strains for each batch of tests. Imipenem-resistant or meropenem-resistant *A. baumannii* complex was considered as CRAB. For analysis of susceptibility rates in different year and patient groups, we used the WHONET software.

### 2.4. Antimicrobial Utilization

We retrospectively obtained the antimicrobial utilization information for all patients by using the hospital pharmacy computer database. The evaluated periods were from 2003 to 2011. Defined daily dose (DDD) was developed by the World Health Organization (WHO) Anatomical Therapeutical Chemical (ATC)/DDD Index 2011 to standardize the comparative usage of various drugs between themselves or between different healthcare environments for all adult wards, and is defined as the assumed average maintenance dose per day for a drug used for its main indication. The amount of the antimicrobials used was calculated as DDD/1,000 patients/day as follows: total consumption measured in DDDs/(number of days in the period of data collection × number of patients) × 1,000 [9]. The six classes of antimicrobial agents analyzed in this study were: anti-pseudomonal penicillins (including mezlocillin, piperacillin, and ticarcillin), β-lactam/β-lactamase inhibitors with anti-pseudomonal effects (ampicillin/sulbactam, cefoperazone/sulbactam, piperacillin/tazobactam, and ticarcillin/clavulanate), anti-pseudomonal cephalosporins (ceftazidime, cefotaxime, ceftriaxone, cefoperazone, and cefepime), anti-pseudomonal carbapenems (imipenem/cilastatin, and meropenem), anti-pseudomonal fluoroquinolones (ciprofloxacin, levofloxacin, and gatifloxacin), and aminoglycosides (amikacin, tobramycin, gentamicin, and netilmicin), modified from suggestion by CLSI in 2011.

### 2.5. Statistical Analysis

Time series analysis model was used to analyze the relationships between the trend in quarterly antimicrobial consumption and the rates of CRAB over time by taking into account the possible time lags (delay for observing an effect of antimicrobial use) and the autocorrelation patterns. AIC were used to check for possible autocorrelation. Logistic regression analysis was performed to analyze the trends in rates of susceptibility of *A. baumannii* to antimicrobials within the study period. All analyses were performed with the Statistical Package for the Social Sciences version 18.0 (SPSS, Chicago, IL, USA). All reported *p* values were two-sided, and values of *p* < 0.05 were considered statistically significant.

## 3. Results and Discussion

### 3.1. Bacterial Isolates

During the 9-year study period, 2,485 consecutive nosocomial isolates of *A. baumannii* complex were isolated in the hospital. Among the isolates, the mean age of the patients was 60.6 ± 18.1 years. The strains were cultured from respiratory samples (1,618 isolates, 65.1%), followed by secretions and pus (465, 18.7%), urine (137, 5.5%), blood (115, 4.6%), pleural fluid and abdominal fluid (104, 4.2%), and others (46, 1.9%). Nine hundred and sixty-four (38.8%) were from intensive care unit (ICU). Table 1 lists the source breakdown of the isolates.

Management of multidrug-resistant *A. baumannii* complex infections is a great challenge for physicians and clinical microbiologists. Its ability to survive in a hospital milieu and its ability to persist for extended periods of time on surfaces makes it a frequent cause for healthcare-associated infections and it has led to multiple outbreaks [13,14]. In humans, *A. baumannii* complex has been isolated from all culturable sites. *A. baumannii* complex can form part of the bacterial flora of the skin, particularly in moist regions such as the axillae, groin, and toe webs, and up to 43.0% of healthy adults can have colonization of skin and mucous membranes, with higher rates among hospital personnel and patients [15]. The most common specimen types in the present study were respiratory samples, secretions and pus over the years in this hospital.

### 3.2. Changes in Resistance to Different Antimicrobial Agents over the Years

Antibiotic susceptibility testing showed that over 40.0% of *A. baumannii* complex isolates were resistant to all 17 antimicrobial agents in 2011. The rates of antimicrobial resistance in *A. baumannii* complex increased significantly in the recent nine years. The resistance rates of *A. baumannii* complex to piperacillin, piperacillin/tazobactam, ticarcillin/clavulanic acid, trimethoprim/sulfamethoxazole, ampicillin/sulbactam, ceftazidime, cefotaxime, ceftriaxone, cefepime and gentamicin were almost more than 60.0%, especially in recent five years. The resistance rates for imipenem, meropenem, amikacin, cefoperazone/sulbactam, ciprofloxacin, levofloxacin and gatifloxacin were shown a relative low, however, they were almost more than 30.0% with a substantial increase during the nine years in the hospital. Resistance to piperacillin, ticarcillin/clavulanic acid, cefoperazone/sulbactam, imipenem, meropenem, cefotaxime, ceftriaxone, amikacin, ciprofloxacin, levofloxacin, and gatifloxacin increased slowly before 2006, then increased significantly thereafter (Table 2). The resistance rate of these antimicrobials did not increase significantly from 2003 to 2006, maybe due to small case number in this period. In contrast, the rates of resistance to piperacillin/tazobactam, ampicillin/sulbactam, ceftazidime, cefepime, and trimethoprim/sulfamethoxazole increased constantly over the years (Table 2).

**Figure 1:**
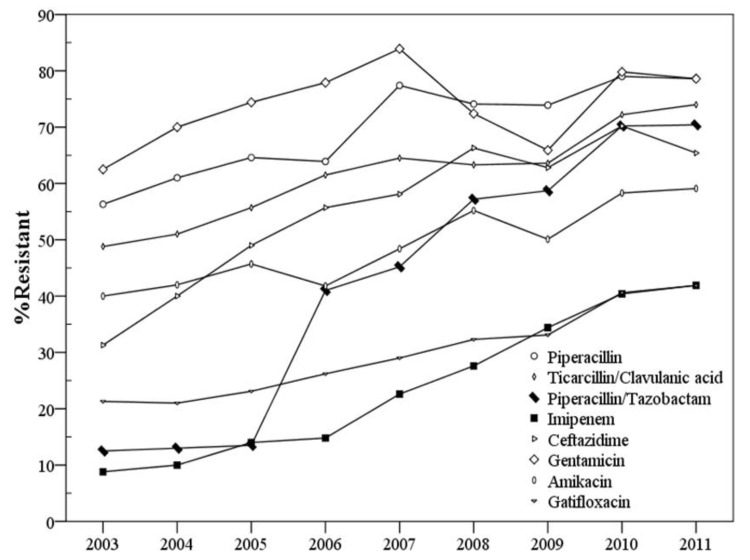
The secular trend of resistance to different antimicrobial agents over the years. Rapid increase of piperacillin/tazobactam resistance (*p* < 0.001; OR, 1.415; 95% CI, 1.358 to 1.475) and imipenem-resistance in *A. baumannii* complex (*p* < 0.001; OR, 1.289; 95% CI, 1.232 to 1.349) compared with the other antimicrobial agents resistance.

The secular trend of resistance to piperacillin, ticarcillin/clavulanic acid, piperacillin/tazobactam, imipenem, ceftazidime, gentamicin, amikacin, and gatifloxacin over the years is shown in Figure 1 to highlight the sharp increase of piperacillin/tazobactam resistance from 12.5% in 2003 to 70.4% in 2011 (*p* < 0.001; odds ratio (OR), 1.415; 95% confidence interval (CI), 1.358 to 1.475) and imipenem resistance from 8.8% in 2003 to 41.9% in 2011 (*p* < 0.001; OR, 1.289; 95% CI, 1.232 to 1.349). The rates of CRAB were 11.3%, 13.0%, 19.8%, 22.1%, 31.0%, 39.7%, 46.5%, 53.5%, and 59.1% between 2003 and 2011. The rates of resistance to different antimicrobial agents between 2003 and 2011 are shown in Table 2.

Due to long-term evolutionary exposure to soil organisms that produce antibiotics, *A. baumannii* complex can develop antibiotic resistance at a much faster pace than other Gram-negative organisms [16]. The emergence of antimicrobial-resistant *A. baumannii* complex is due both to the selective pressure exerted by the use of broad-spectrum antimicrobials and transmission of strains among patients, although the relative contributions of these mechanisms are not yet known [17]. Carbapenems such as imipenem and meropenem are the last resort of drugs for the treatment of multidrug-resistant pathogens including *A. baumannii* complex. However, the incidence of carbapenem resistance in *A. baumannii* complex increased steadily in the 2000s [7,18]. This study of 2,485 *A. baumannii* complex over the years revealed the continuous increase of antimicrobial resistance. Resistance to carbapenems, which is often accompanied with resistance to multiple other agents, has increased in all parts of the world. Our study revealed the rapid increase in the prevalence of CRAB over the years the hospital, from 11.3% in 2003 to 59.1% in 2011. The results of the present study indicate a strong burden of CRAB in the hospital.

**Table 1:** Source breakdown of *A. baumannii* complex isolated from First Hospital of Jilin University, 2003–2011.

Strata		2003		2004		2005		2006		2007		2008		2009		2010		2011		Total	
		*n*	%	*n*	%	*n*	%	*n*	%	*n*	%	*n*	%	*n*	%	*n*	%	*n*	%	*n*	%
*Patient location*																					
	ICU	25	31.3	32	32.0	39	36.8	47	38.5	60	38.7	115	38.7	155	40.1	179	40.4	312	39.2	964	38.8
	Non-ICU	55	68.8	68	68.0	67	63.2	75	61.5	95	61.3	182	61.3	232	59.9	264	59.6	483	60.7	1521	61.2
*Specimen type*																					
	Respiratory	52	65.0	65	65.0	68	64.2	79	64.8	100	64.5	194	65.3	258	66.7	287	64.8	515	64.8	1618	65.1
	Secretions and pus	15	18.8	18	18.0	20	18.9	23	18.9	28	18.1	56	18.9	70	18.1	89	20.1	146	18.4	465	18.7
	Urine	5	6.3	5	5.0	6	5.7	7	5.7	9	5.8	16	5.4	19	4.9	22	5.0	48	6.0	137	5.5
	Blood	4	5.0	5	5.0	5	4.7	6	4.9	9	5.8	13	4.4	18	4.7	19	4.3	36	4.5	115	4.6
	Pleural fluid and abdominal fluid	2	2.5	4	4.0	5	4.7	5	4.1	6	3.9	14	4.7	17	4.4	18	4.1	33	4.2	104	4.2
	Others	2	2.5	3	3.0	2	1.9	2	1.6	3	1.9	4	1.3	5	1.3	8	1.8	17	2.1	46	1.9
*Total*		80		100		106		122		155		297		387		443		795		2485	

**Table 2:** Antimicrobial resistance of *A. baumannii* complex isolated from First Hospital of Jilin University, 2003–2011.

Antimicrobial agents	Resistance rate (%) by year									2003 to 2006			2006 to 2011		
	2003 (*n* = 80)	2004 (*n* = 100)	2005 (*n* = 106)	2006 (*n* = 122)	2007 (*n* = 155)	2008 (*n* = 297)	2009 (*n* = 387)	2010 (*n* = 443)	2011 (*n* = 795)	*p*	OR	95% CI	*p*	OR	95% CI
Piperacillin	56.3	61.0	64.6	63.9	77.4	74.1	73.9	79.0	78.6	0.274	1.379	0.775–2.453	0.001	2.074	1.381–3.114
Ticarcillin/Clavulanic acid	48.8	51.0	55.7	61.5	64.5	63.3	63.6	72.2	74.0	0.075	1.678	0.949–2.967	0.004	1.780	1.196–2.649
Piperacillin/Tazobactam	12.5	13.0	13.5	41.0	45.2	57.2	58.7	70.2	70.4	0.001	4.861	2.286–10.337	0.001	3.431	2.319–5.077
Cefoperazone/Sulbactam	13.8	13.0	13.2	20.5	43.9	27.9	40.1	40.6	44.7	0.223	1.617	0.746–3.504	0.001	3.130	1.974–4.965
Ampicillin/Sulbactam	37.5	39.0	44.4	61.0	71.0	69.0	63.6	73.4	75.6	0.001	2.569	1.438–4.591	0.001	2.009	1.350–2.991
Imipenem	8.8	10.0	14.0	14.8	22.6	27.6	34.4	40.4	41.9	0.210	1.805	0.717–4.542	0.001	4.165	2.476–7.003
Meropenem	7.5	9.0	9.6	15.6	23.2	27.3	39.5	39.5	40.3	0.095	2.275	0.867–5.973	0.001	3.652	2.194–6.079
Ceftazidime	31.3	40.0	49.0	55.7	58.1	66.3	62.8	70.2	65.4	0.001	2.770	1.532–5.011	0.039	1.502	1.021–2.209
Cefotaxime	45.0	50.0	56.6	59.0	63.9	68.7	71.1	74.5	76.7	0.052	1.760	0.996–3.110	0.001	2.290	1.540–3.404
Ceftriaxone	48.8	51.0	59.6	60.7	65.2	71.7	70.8	75.0	76.7	0.096	1.621	0.917–2.864	0.001	2.139	1.435–3.187
Cefepime	28.8	30.0	34.0	52.5	58.1	68.0	61.2	70.2	66.0	0.001	2.735	1.500–4.986	0.004	1.762	1.200–2.588
Gentamicin	62.5	70.0	74.4	77.9	83.9	72.4	65.9	79.8	78.6	0.030	2.000	1.071–3.734	0.678	1.103	0.695–1.751
Amikacin	40.0	42.0	45.7	41.8	48.4	55.2	50.1	58.3	59.1	0.799	1.077	0.607–1.913	0.001	2.013	1.368–2.963
Ciprofloxacin	28.8	31.0	33.3	37.7	45.2	55.2	46.5	60.4	59.7	0.190	1.500	0.817–2.752	0.001	2.452	1.656–3.632
Levofloxacin	19.0	19.0	22.6	31.1	39.4	47.1	36.4	53.5	56.6	0.052	1.960	0.993–3.868	0.001	2.883	1.917–4.336
Gatifloxacin	21.3	21.0	23.1	26.2	29.0	32.3	33.1	40.6	41.9	0.420	1.318	0.674–2.576	0.001	2.027	1.322–3.108
Trimethoprim/Sulfamethoxazole	47.5	49.0	51.9	64.8	70.2	70.4	64.9	74.4	77.4	0.016	2.031	1.143–3.608	0.003	1.860	1.238–2.794

**Table 3:** Correlation between quarterly consumption of antimicrobial agents and rates of CRAB in First Hospital of Jilin University, 2003–2011.

Antimicrobial agents			Penicillins	β-lactam/β-lactamase inhibitors	Cephalosporins	Carbapenems	Aminoglycosides	Fluoroquinolones
**Antimicrobial consumption (DDDs/1,000 patients/day) by quarter**	**2003**	**1st quarter**	1.8	73.7	139.8	3.1	44.1	46.7
		**2nd quarter**	1.9	76.6	143.7	3.2	44.1	43.4
		**3rd quarter**	1.9	75.9	141.6	3.2	43.8	45.1
		**4th quarter**	1.8	75.1	139.1	3.4	43.8	44.1
	**2004**	**1st quarter**	1.7	146.2	123.6	4.9	44.8	49.9
		**2nd quarter**	1.7	147.5	117.8	5.1	44.1	53.1
		**3rd quarter**	1.6	159.5	120.1	5.1	43.9	51.1
		**4th quarter**	1.6	169.3	112.3	5.3	44.8	52.3
	**2005**	**1st quarter**	1.6	217.9	59.3	6.9	45.3	63.8
		**2nd quarter**	1.7	289.3	53.1	7.6	44.8	65.7
		**3rd quarter**	1.7	300.9	50.1	8.1	43.9	69.7
		**4th quarter**	1.8	278.9	48.2	8.2	44.6	68.6
	**2006**	**1st quarter**	1.5	180.5	92.4	8.5	43.8	50.3
		**2nd quarter**	1.2	170.5	103.8	8.8	42.3	49.8
		**3rd quarter**	1.3	161.2	120.4	9.1	42.1	50.6
		**4th quarter**	1.5	160.7	129.5	9.9	43.2	49.1
	**2007**	**1st quarter**	2.1	120.3	147.8	10.9	39.7	41.8
		**2nd quarter**	2.7	112.2	159.3	11.9	40.9	42.1
		**3rd quarter**	2.9	101.1	172.6	12.6	41.6	39.1
		**4th quarter**	2.9	91.2	165.1	13.1	40.6	39.5
	**2008**	**1st quarter**	3.9	59.8	151.7	13.9	35.6	36.3
		**2nd quarter**	4.4	60.1	140.7	14.1	33.4	37.2
		**3rd quarter**	4.9	52.1	136.7	14.5	30.9	31.4
		**4th quarter**	4.8	54.2	130.9	15.1	31.3	31.3
	**2009**	**1st quarter**	3.1	80.3	189.9	23.9	38.3	43.2
		**2nd quarter**	3.0	76.9	198.8	26.6	36.7	43.6
		**3rd quarter**	2.1	79.4	195.4	25.4	35.6	43.6
		**4th quarter**	2.4	75.1	190.1	25.0	38.1	42.9
	**2010**	**1st quarter**	0.8	59.3	146.7	27.5	69.8	30.3
		**2nd quarter**	0.4	56.7	151.2	28.5	77.7	27.4
		**3rd quarter**	0.4	52.1	140.1	28.9	78.8	31.2
		**4th quarter**	0.3	53.9	138.5	28.1	73.6	27.1
	**2011**	**1st quarter**	15.2	69.6	157.3	29.9	25.5	40.8
		**2nd quarter**	16.1	71.9	149.4	29.1	25.1	46.3
		**3rd quarter**	16.7	74.2	159.2	30.4	24.1	45.9
		**4th quarter**	15.8	76.1	145.2	29.9	23.9	44.2
**Time-series analysis model**		**β**	0.199	−0.015	−0.021	1.661	−0.001	−0.010
		***p***	0.473	0.546	0.561	0.001	0.987	0.936

### 3.3. Association of Hospital Antimicrobial Usage and the Rates of CRAB

Overall, the consumption of anti-pseudomonal carbapenems significantly increased during the 9-year study period. In contrast, the annual use of β-lactam/β-lactamase inhibitors with anti-pseudomonal effect, and fluoroquinolones significantly decreased. Use of anti-pseudomonal aminoglycosides, and cephalosporins remained stable, whereas the penicillins use fluctuated over the years (Table 3). The association between the rates of CRAB and quarterly consumption of antimicrobial agents of different classes from 2003 to 2011 are shown in Table 3.

Table 3 shows correlation between quarterly consumption of antimicrobial agents used for treatment of infections due to *A. baumannii* complex and rates of CRAB. The results indicate that the quarterly use of anti-pseudomonal carbapenems was strongly correlated with the increase of quarterly CRAB (β = 1.661; *p* < 0.001). None of the other classes of antimicrobial agents was significantly associated with the increase in quarterly CRAB (Table 3).

Previous studies on the influence of antibiotic exposure on the risk for acquiring CRAB demonstrated that prior usage of carbapenem [8,19,20], and cephamycin [21] might play a role. Carbapenems may be considered the treatment of choice for empirical treatment of patients with ESBL-producing *Enterobacteriaceae bacteraemia* in China. This followed an outbreak of CRAB infections due to ESBL-producing Enterobacteriaceae during which use of carbapenems increased substantially in China. We found that prior exposure of imipenem and meropenem was associated with CRAB acquisition in this study. Imipenem and meropenem are broad-spectrum antibiotics with activities against most Gram-negative bacteria, including many nonfermentative Gram-negative bacilli. Therefore, it is understandable that carbapenems usage could change the bacterial flora in patients and facilitate the colonization and/or infection of resistant bacteria, such as CRAB. We found that the quarterly use of anti-pseudomonal carbapenems was strongly correlated with the increase of quarterly CRAB; however, none of the other classes of antimicrobial agents was significantly associated with the increase in quarterly CRAB.

Environmental contamination was also found to be important in the outbreaks of CRAB. Implicated items included respiratory equipment, ventilator tubing, suctioning equipment, bed rails, curtains, ambu bags, washbasins, trunking, peak flow meters, intravenous catheters, *etc.* [22]. Contaminated hands of healthcare workers were found to be involved in a significant number of cases [22]. It is obvious that multiple factors result in the outbreaks of CRAB in hospital. 

The limitation of this paper is that we have no pulse field electrophoresis data of *A. baumannii* complex. We could not determine the outbreak of *A. baumannii* complex in different wards. Our research is an ecological study that use aggregated population-level data. Indeed, multilevel analysis makes possible the exploration of joint effects of exposures to antimicrobial agents at individual level and group level on the acquisition of resistant bacteria.

## 4. Conclusions

Thus, to decrease the spread of *A. baumannii* complex infections and reduce the pace of emergence of resistance in multidrug-resistant (MDR) *A. baumannii* complex, it is important to promote the rational use of antimicrobials with microbiology laboratory support in hospitals. Dedicated use of anti-pseudomonal carbapenems would be an important intervention to control the increase of CRAB. Hand hygiene and barrier nursing are important to keep the spread of infection in check. Surveillance is therefore important in providing useful information for physicians in choosing empirical antibiotics. It also helps to address specific resistant issues within a region to help identify targeted intervention measure.
